# Establishment and characterization of a cell line (OS-MM) originating from a human malignant melanoma of the oral mucosa

**DOI:** 10.1007/s11626-026-01162-2

**Published:** 2026-02-21

**Authors:** Tomoaki Shintani, Atsuko Hamada, Sachiko Yamasaki, Yukari Jono, Koichi Koizumi, Ryouji Tani, Akihiko Sakamoto, Yasusei Kudo, Mikihito Kajiya, Souichi Yanamoto, Tetsuji Okamoto

**Affiliations:** 1 Center of Oral Clinical Examination, Hiroshima University Hospital, Hiroshima, 734-8553 Japan; 2 Department of Oral Oncology, Graduate School of Biomedical, and Health Sciences, Hiroshima University, Hiroshima, 734-8553 Japan; 3 School of Medical Sciences, University of East Asia, Shimonoseki, 751-8503 Japan; 4 Oral Maxillofacial Surgery, Chugoku Rosai Hospital, Kure, 737-0193 Japan; 5 Oral Maxillofacial Surgery, Mazda Hospital, Hiroshima, 735-8585 Japan; 6 Department of Oral Bioscience, Tokushima University Graduate School of Biomedical Sciences, Tokushima University, Tokushima, 770-8504 Japan

**Keywords:** Cell line, Malignant melanoma, Oral mucosal melanoma, Molecular genetics, Vascular endothelial growth factor (VEGF), Angiogenesis, VEGF receptor (VEGFR)

## Abstract

The prognosis for patients diagnosed with oral mucosal melanoma is poor, and the etiology of this disease remains to be elucidated. The underlying reason for this is that no malignant melanoma (MM) cell line derived from oral mucosal tissue has been successfully established to date. The establishment of a human MM cell line designated OS-MM from a tissue sample of a patient diagnosed with palatal mucosal melanoma was undertaken. For a period exceeding three decades, tumor cells have undergone uninterrupted proliferation, exhibiting a spindle-like morphology. Chromosomes exhibit a low-triploid pattern with an observed mode of 47. Heterotransplantation of the cells into SCID mice has resulted in the formation of tumor masses. The cells expressed vascular endothelial growth factor (VEGF) and its receptors. The addition of exogenous recombinant VEGF_165_ did not alter proliferation but enhanced motility. A genetic analysis of OS-MM cells revealed the absence of BRAF and NRAS mutations; however, a GNAS mutation was identified. The OS-MM cell line should contribute to advances in personalized therapy for malignant oral mucosal melanoma.

**Supplementary Information:**

The online version contains supplementary material available at 10.1007/s11626-026-01162-2.

## Introduction

Malignant melanoma (MM) is widely recognized as the most poorly prognostic malignant tumor of neuroectodermal origin due to its rapid progression and its early hematogenous or lymphogenous metastasis (Fallarino and Blank 2025; Tarnawski *et al*. 2025). Primary MM of the head and neck mucosa is a rare malignant tumor, first reported by Weber in 1859 (Gondivkar *et al*. 2009). The etiology of this tumor is attributed to the uncontrolled proliferation of atypical melanocytes, neural crest-derived cells, and melanin-producing cells (Kauzman *et al*. 2004; Chatzistefanou *et al*. 2016; López *et al*. 2016). The most frequently impacted sites within the oral cavity include the hard palate and maxillary gingiva, followed by the buccal mucosa, lips, tongue, and floor of the mouth (López *et al*. 2016). In general, oral mucosal MM is asymptomatic in its early stages. However, the disease is often diagnosed at an advanced stage, resulting in a low 5-yr survival rate of 15% (Hicks and Flaitz 2000; López *et al*. 2016; Panda *et al*. 2018). The therapeutic approach to oral mucosal MM is contingent upon the tumor stage and location with surgical intervention or its combination with chemotherapy being the prevailing modalities (Kauzman *et al*. 2004; López *et al*. 2016; Yde *et al*. 2018). As demonstrated in the study by Bansal *et al*. (2022), the use of radiation therapy and chemotherapy as standalone treatments was shown to be ineffective (Bansal *et al*. 2022). Immunological techniques have undergone substantial development, and the relationship between MM and the immune system has been thoroughly investigated. It has been documented that MM evades the immune system during the formation of tumors, and the presence of the melanoma-associated antigen has been identified (Gide *et al*. 2018; Xiong and Cheng 2025). Despite the development of numerous therapeutic strategies, most have failed to meaningfully improve the prognosis of MM patients. The survival rates for MM patients are lower than for cutaneous melanoma patients, the majority of whom are currently diagnosed at an early stage. Oral mucosal melanoma has been shown to be much more aggressive than cutaneous melanoma. Furthermore, the prognostic markers for MM have not yet been fully elucidated (López *et al*. 2016). Regarding MM cell lines, the preponderance of those studied is derived from skin, with no examples derived from oral mucosa (Carey *et al*. 1979; Reichard-Brown and Akeson 1983; Sohmura *et al*. 1986). Consequently, the establishment of the human MM cell line as in vitro models for studying tumor biology, including drug sensitivity, is imperative.

Angiogenic factors such as fibroblast growth factor-2 (FGF-2) (Gospodarowicz 1987) and vascular endothelial growth factor (VEGF) (Myoken *et al*. 1991), which is also known as a vascular permeability factor (VPF) (Senger *et al*. 1986), are well known to stimulate tumor angiogenesis and neovascularization. Angiogenic factors secreted by malignant tumor cells act to target cells in autocrine or paracrine mechanisms (Myoken *et al*. 1994). FGF-2 and VEGF have been identified as critical players in the angiogenic factors secreted by malignant tumors. As we previously indicated, A431 vulvar carcinoma cells have been demonstrated to secrete VEGF. This factor has been shown to bind to the binding sites of the human umbilical vein endothelial cells (HUVEC), thereby stimulating their proliferation (Myoken *et al*. 1991).

This study presents the establishment and characterization of a novel human MM cell line designated as an OS-MM originating from a human MM of the oral mucosa. Utilizing the cells, we performed in vitro proliferation capacity, chromosome analysis, and a thorough investigation into the expression and function of VEGF and VEGFRs. Additionally, we assessed their tumorigenicity in nude mice. Furthermore, immunohistochemical analysis of the cells and xenografted tumors was conducted. Additionally, an analysis was performed on the involvement of 88 genes implicated in tumorigenesis and tumor biology in the cells.

## Material and methods

### Patients history

The patient was a 68-yr-old woman who first presented to the Hiroshima University Dental Hospital in April 1983 with a chief complaint of a mass formation on the palatal mucosa and bleeding from that area. The patient exhibited no prior history of smoking or excessive alcohol consumption. Her family history was unremarkable. The patient’s medical history includes a history of gastric ulcers and hypertension. Intraoral examination revealed an elastic hard and dark red mass on the right palatal gingiva. The clinical diagnosis was primary malignant melanoma of the maxillary mucosa (cT2N0M0) stage II. A right partial maxillectomy was performed after induction chemotherapy in June 1983. Subsequent outpatient follow-ups revealed tumor re-growth in the palate and alveolar mucosa of the maxillary anterior region in February 1991. Subsequent tumor resection was performed. This cell line was established from tissue samples obtained during this surgery.

### Primary culture

Primary cell culture was initiated from tissue specimens obtained during the 1991 tumor resection. Following this, the specimen underwent a thorough cleansing process involving a single cycle of washing with 70% ethanol and two subsequent cycles of washing with phosphate-buffered saline (PBS, Sigma-Aldrich, St. Louis, MO). The specimen was then subjected to a mechanical mincing procedure, employing sterile blades of No. 15 (FEATHER, Osaka, Japan). The cells were cultured in a 1:1 mixture of Dulbecco’s modified Eagle’s medium and Ham F-12 Medium (Wako, Osaka, Japan) supplemented with 1% calf serum (CS, Global Life Sciences Technologies, Tokyo, Japan) and six factors: insulin (10 µg/mL), transferrin (5 µg/mL), 2-aminoethanol (10 µM), sodium selenite (10 nM), 2-mercaptoethanol (10 µM), and oleic acid conjugated with fatty acid-free bovine serum albumin (9.4 µg/mL) (Sato *et al*. 1987; Okamoto *et al*. 1996; Rosli *et al*. 2014; Shintani *et al*. 2016). We designated the serum-free medium as DF6F. All chemicals were from Sigma-Aldrich. All reagents used for cell culture were free of mycoplasma and viral pathogens. Cells were cultured in 60-mm culture dishes (BD Falcon, San Jose, CA) at 37 °C in a humidified 95% air/5% CO_2_ atmosphere in a CO_2_ incubator (Thermo Fisher Scientific, Waltham, MA) until reaching 70–80% confluence. The cells were observed with a phase contrast microscope (Nikon Co., Tokyo, Japan). The cells were subsequently subjected to trypsin-ethylenediaminetetraacetic acid (EDTA) (Sigma) treatment for the subculture. The established cell line was designated OS-MM, and it has been successfully maintained for more than 20 yr. The authentication of these cells was conducted using mitochondrial DNA (mtDNA) analysis as described below, and the short tandem repeat (STR) profiling (BEX Co., Ltd., Tokyo, Japan) (Table 1). Recombinant human vascular endothelial growth factor (rVEGF_165_,165 amino acid form) was obtained from Upstate Biotechnology, Inc. (Lake Placid, NY). Na[^125^I] was obtained from New England Nuclear (Boston, MA). The molecular weight molecular marker was obtained from Bio-Rad Laboratories (Hercules, CA).

**Table 1.** STR profile of OS-MM cell line

D3S1358	TH01	D21S11	D18S51	Penta_E	D5S818	D13S317	D7S820
15	9	29	12	15	10	11	11
		31	13	23	12	13	
D16S539	CSF1PO	Penta_D	AMEL	vWA	D8S1179	TPOX	FGA
10	12	9	X	14	13	11	19
	13	11		18		12	

### Mitochondrial DNA (mtDNA) isolation and sequencing analysis

The extraction and purification of mtDNA from the OS-MM cells and surgical tissue were carried out by an external contractor (Fasmac Co., Ltd., Kanagawa, Japan). To facilitate personal identification, DNA sequences were ascertained from position 16024 to 16365 in mtDNA hypervariable region 1 (HV1) and from 73 to 340 in mtDNA hypervariable region 2 (HV2) (Fasmac Co., Ltd.) (Imaizumi *et al*. 2002). Informed consent was obtained from the bereaved family, including consent for participation and publication of the findings. The study was conducted in accordance with the ethical committee of Hiroshima University Hospital (approval number: hi-72).

### Growth characteristics

To investigate cell proliferation in serum-free defined culture, 1 × 10^4^ OS-MM cells were seeded into a 24-well culture plate (BD Falcon, San Jose, CA) in DF5F and DF6F medium. The cell count was performed daily for a period of 6 d using a Coulter counter (Beckman Coulter, Tokyo, Japan) daily. Next, 1 × 10^4^ OS-MM cells were seeded into a 24-well culture plate (BD Falcon, San Jose, CA), in culture medium consisted of DF nutrient medium alone and DF6F supplemented with various concentrations of calf serum (CS, Global Life Sciences Technologies, Tokyo, Japan). The cell count was performed after 8 d culture using a Coulter counter.

### Tumorigenicity in SCID mice

Three 4-wk-old male athymic Balb/c severe combined immunodeficient (SCID) mice (Charles River Japan, Tokyo, Japan) were subcutaneously (s.c.) inoculated into the back. The injection of 0.1 mL of PBS containing 10 million OS-MM cells was performed. The mice were observed on a weekly basis to monitor the development of xenografts. Thirty-two weeks after injection, the subcutaneous tumors had attained a diameter of 8–10 mm. The mice were euthanized, and the subcutaneous tumors were surgically removed and placed in 10% PBS-buffered formalin (Sigma) for subsequent histological and immunohistochemical analysis.

### Immunohistochemical analysis

Tissues were fixed in 10% PBS-buffered formalin overnight at ambient temperature for a 12-h period. Subsequently, they were processed into blocks using a paraffin embedding technique. Paraffin-embedded tissues were sectioned at a thickness ranging from 4 to 6 μm for subsequent immuno-staining procedures. The sections were deparaffinized in xylene (Wako), dehydrated with alcohol, and rehydrated in PBS. The activity of endogenous peroxidase was blocked with 3% hydrogen peroxide (Wako) in methanol (Sigma). One thousand cells/chamber of OS-MM cells cultured in DF6F on the LAB-TEK Chamber Slide (Nulgen Nunc International, Naperville, IL) were fixed with 4% paraformaldehyde for 20 min, washed twice with PBS, and blocked with 1% bovine serum albumin (BSA) in PBS for 30 min at room temperature. The sections and the cells were incubated overnight at 4 °C with a series of primary antibodies, including mouse monoclonal anti-Melanosome antibody (HMB-45; 1:100; DAKO A/S, Copenhagen, Denmark), rabbit polyclonal anti-S-100 antibody (DAKO A/S), mouse monoclonal anti-Mart-1/Melan-A antibody (M2-7C10; Nichirei Biosciences, Inc., Tokyo, Japan), rabbit polyclonal anti-keratin antibody (DAKO A/S), rabbit polyclonal anti-glial fibrillary acidic protein (GFAP) antibody (DAKO A/S), and rabbit polyclonal anti-vimentin antibody (DAKO A/S). The sections were then exposed to peroxidase-conjugated secondary antibody (1:100; DAKO) for 1 h, and positive staining was detected with 3,3-diaminobenzidine (DAKO). The slides were stained with hematoxylin and eosin (HE, Sigma) and mounted using Mount-Quick (Fisher Scientific, Houston, TX). The images were captured using the EVOS FL Auto 2 Imaging System (product #AMAFD2000) with an Olympus Super Apochromat objective (product #AMEP4754) and a Nikon Eclipse E800 microscope. The subsequent analysis of the images was conducted using Adobe Photoshop Elements (Adobe Inc., San Jose, CA).

### Chromosome analysis

The identification of the karyotype was investigated by means of the method developed by Moorhead (Moorhead 1965). Tumor cells were cultivated in 60-mm plastic dishes (BD Falcon) prior to achieving confluency. Following the addition of Colcemid (10 µg/mL) (Nakarai Tesque, Inc., Kyoto, Japan) at a final concentration of 0.25 µg/mL, the cells were subjected to a 6-h culture period. Subsequently, the melanoma cells were harvested through a trypsin-EDTA treatment. Following a centrifugation process at 1200 rpm for a duration of 5 min at ambient temperature, the cells were subjected to an incubation with 0.075 M KCL (Wako) at 37 °C for a period of 30 min, which induced cell lysis. The cells were meticulously prepared for analysis by subjecting them to a fixation process involving a solution composed of a three-to-one ratio of ethanol to acetic acid. Following this, the cells were released from their confining medium and positioned at a precise distance from the surface of the slide glass. The fixation process was then followed by a drying procedure that utilized the natural air circulation to facilitate the drying process. The slide glasses were stained with Giemsa stain (Beckman Coulter). The chromosome number was determined through the analysis of 78 individual cells.

### The level of 5-S-cysteinyldopa (5-S-CD) in culture medium

The level of 5-S-cysteinyldopa (5-S-CD), a common product of malignant melanomas, present in the culture medium of the cells was determined through the application of high-performance liquid chromatography with electrochemical detection (SRL, Inc., Tokyo, Japan). The cells were cultured in DF6F medium supplemented with 2% CS. Medium was collected after 2 and 5 d of culture and used as samples. Medium supplemented with 2% CS in DF6F was used as a control.

### Radioactive iodine labeling of rVEGF_165_

The radioactive iodination of rVEGF_165_ was performed using the modified chloramine T method (Kan *et al*. 1988). The specific activity of labeled rVEGF_165_ was 1.5 × 10^5^ cpm/ng.

### Receptor binding assay

The OS-MM cells were seeded in 48-well plastic plates (BD Falcon) in DF6F medium supplemented 1% CS at the density of 1.0 × 10^4^ cells/well. On the second day, the OS-MM cells were washed three times with DF buffer (pH 7.4) containing 25 mM HEPES (Dojindo Laboratories, Kumamoto, Japan) and 0.1% BSA. The cells were then placed in a 37 °C incubator for 2 h. Following the preincubation period, various doses of labeled rVEGF_165_ were added to the cells in each well, which was then incubated for 4 h on ice for facilitate binding. Subsequently, the OS-MM cells were washed three times with DF buffer. The membrane fraction of the cells was solubilized with 100 mL of 1% Triton-X100 (Sigma) in distilled water for 30 min at room temperature. The radioactivity of the aliquot was measured using an Autowell g-counter (Aloka, Tokyo, Japan). The estimation of nonspecific binding was derived from the quantification of cell-associated radioactivity in the presence of a 100-fold molar excess of unlabeled rVEGF_165_.

### RNA extraction and RT-PCR for VEGFRs mRNAs

The expression levels of VEGF-A, VEGFR1/flt-1, and VEGFR2/KDR were verified through RT-PCR analysis. The primer sequences are listed in Table 2. Experimental methods were as previously described by Koizumi *et al*. (2022).

**Table 2.** Oligonucleotide primer sequences

Genes	Primer sequences
GAPDH	F-5′-GCTCTCTGCTCCTCCTGTTC-3′
	R-5′-ACGACCAAATCCGTTGACTC-3′
VEGF-A	F-5′-CTTGCCTTGCTGCTCTACC-3′
	R-5′-CACACAGGATGGCTTGAAG-3′
VEGFR1/flt-1	F-5′-GTCACAGAAGAGGATGAAGGTGTCTA-3′
	R-5′-CACAGTCCGGCACGTAGGTGATT-3′
VEGFR2/KDR	F-5′-CCTGTCCACTTACCTGAGGAG-3′
	R-5′-CTGGCTACTGGTGATGCTGTC-3′

### Cell motility assay

The analysis of cell motility was conducted using a modified Boyden chamber assay with Transwell inserts (6.5 mm diameter) with 8-μm pores (Coaster, Cambre, MA), as previously described by Hayashido *et al*. (2007) and Higaki *et al*. (2020). OS-MM cells (5 × 10^5^) were seeded in DF nutrient medium containing 0.1% BSA to the upper compartment of each Transwell insert. Following a 24-h culture at 37 °C, the Transwell inserts were fixed with methanol and stained with Diff-Quick (Dade Behringen, Duedingen, Switzerland). The cells located on the anterior surface of the filter were meticulously transferred using a cotton swab. The cells that migrated on the back surface of the filter were stained, and cell numbers were counted using light microscopy under a high-power field (× 200). The cell numbers in the four fields were enumerated in each of the three distinct experiments. The results were expressed as the mean number of migrating cells/mm^2^ ± standard deviation (SD), indicated as a measurement of variability.

### DNA isolation and gene panel sequencing analysis

The extraction and purification of genomic DNA from the OS-MM cells was performed by an external contractor (Macrogen, Tokyo, Japan). A subsequent analysis of the genes listed in Table S1 (single nucleotide variants (SNVs)/insertion‑deletions (InDels) (88 genes) and fusions (3 genes)) was performed using next‑generation sequencing with the Axen™ Cancer Panel 1 (Macrogen). Informed consent was obtained from the bereaved family, including consent for participation and publication of the findings. The study was conducted in accordance with the ethical principles established by the Hiroshima University Hospital ethical committee (approval number: hi-72).

### Statistical analysis

Statistical analyses were conducted using JMP Pro 16 (SAS Institute Inc., Cary, NC). All data were presented as the mean ± standard deviation (SD) of at least three independent experiments. The Student’s *t*-test was used to compare the difference between two groups. The differences were considered to be significant at *p* < 0.05.

## Results

**Figure 1.**
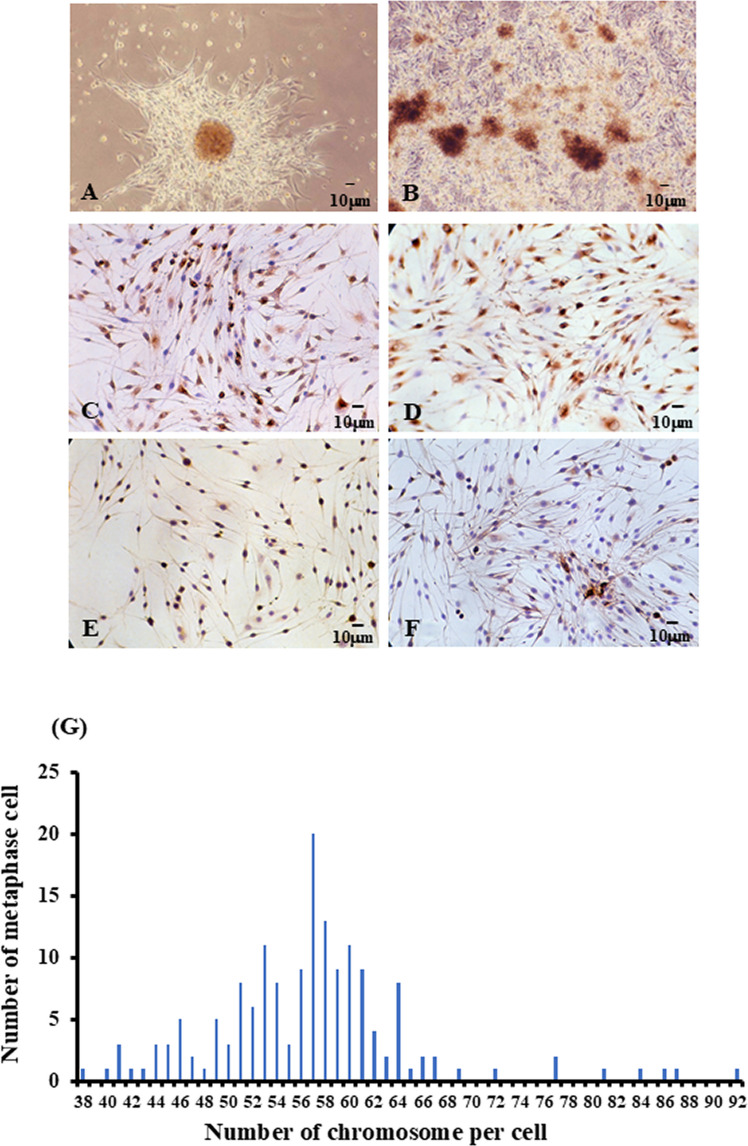
Morphologic, immunohistochemical, and chromosomal features of OS-MM cells. Cultured cells were obtained with a phase contrast microscope (× 40) at primary culture (*A*, *B*). Immunohistochemical staining of the cells revealed that HMB-45 (*C*), GFAP (*D*), keratin (*E*), and vimentin (*F*) were all positive in OS-MM cells. The distribution of chromosome number in OS-MM cells is shown, and the most prevalent chromosome number was identified as 57 (range 38 to 92) (*G*).

**Figure 2.**
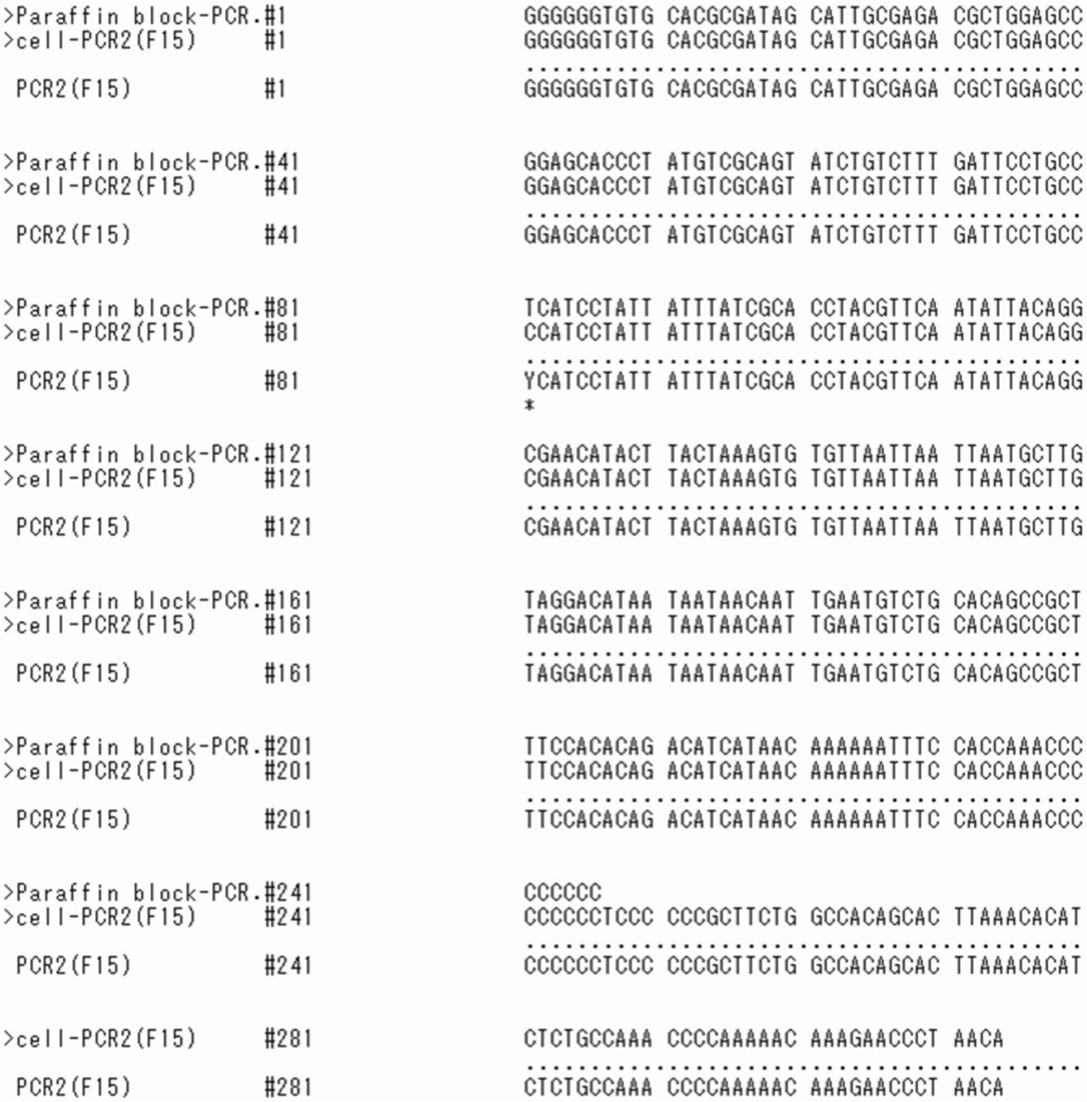
Sequence alignment of the mtDNA HV2 region in OS-MM cells and the patient tissue. Mitochondrial DNA (mtDNA) was extracted from the paraffin blocks of the surgical specimens and cultured cells, and sequence analysis of the FV2 region was performed

**Figure 3.**
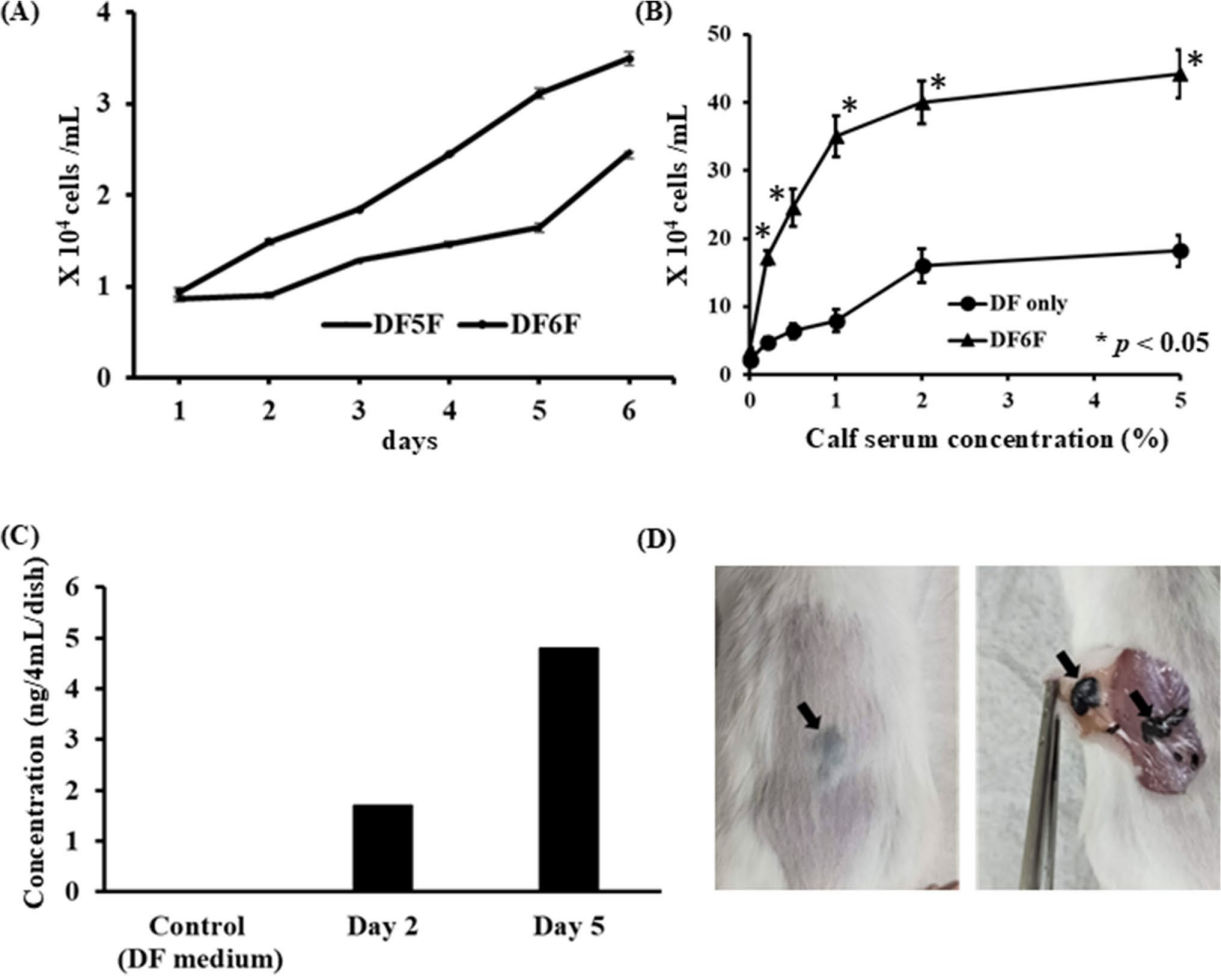
In vitro proliferation of OS-MM cells, the quantification of 5-S-CD in culture medium, and assessment of tumor formation in SCID mice. The proliferation of OS-MM cells in DF5F and DF6F was demonstrated (*A*). It is shown that OS-MM cells are less serum-dependent in DF6F medium (*B*). Data are presented as mean tumor volume ± SD. The concentration of 5-S-CD in the OS-MM cell culture medium was subsequently quantified (*C*). The tumor produced by heterotransplantation of OS-MM cells into SCID mice was macroscopically observed (*D*).

The OS-MM cell line, which was derived from an oral mucosal MM, was established as described above and thoroughly characterized. The proliferation of tumor cells with a spindle-like morphology has been stable for a period exceeding three decades (Fig. 1*A*, *B*). Under culture conditions using DF6F medium supplemented with 2% CS, the doubling time of OS-MM cells was 26.6 h (data not shown). Immunohistochemical staining of the cells revealed that they expressed HMB-45, GFAP, keratin, and vimentin (Fig. 1*C*–*F*). The modal chromosome number was 57 (range 38 to 92), which suggested that these melanoma cells exhibited a sub-triploid pattern (Fig. 1*G*). The nucleotide sequences of the HV2 region in mtDNA extracted from the cells and the tissues were found to be identical (Fig. 2). The OS-MM cells proliferated in both DF5F and DF6F serum-free defined medium, but proliferation in DF6F was superior to that in DF5F (Fig. 3*A*). Concurrently, the proliferation capacity and saturation density of OS-MM cells were examined in DF nutrient medium or DF6F serum-free medium in the presence of various serum concentrations. The results demonstrated that the saturation density of the cells in DF6F was significantly higher than that in DF nutrient medium (Fig. 3*B*, *p* < 0.05). In addition, the concentration of 5-S-CD in the culture medium exhibited an increase on the second and fifth days (Fig. 3*B*).

**Figure 4.**
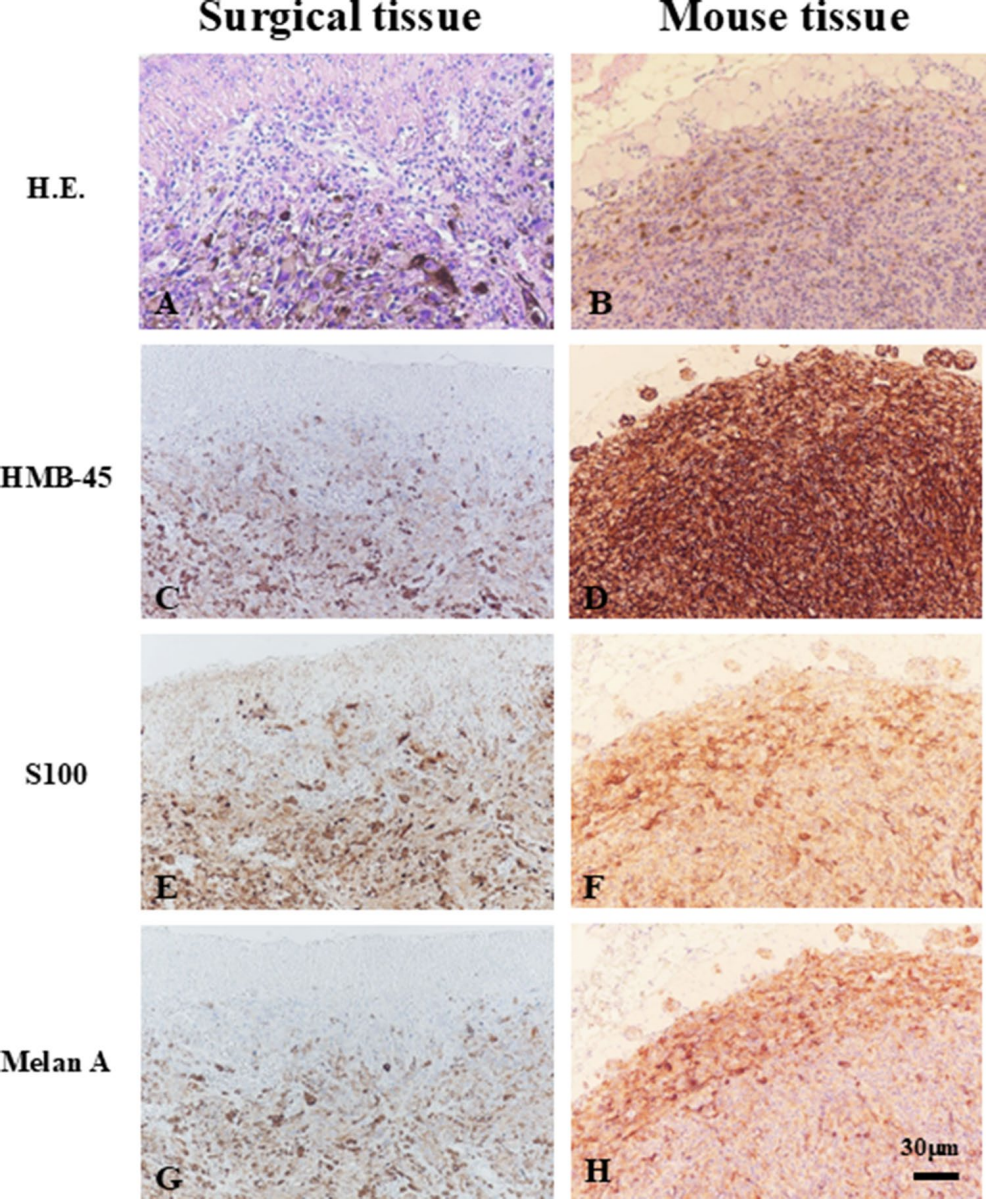
Histopathological features of the OS-MM cells in the surgical tissue and mouse-derived tumor. The surgical tissue (*A*) and the mouse tissue (*B*) were subjected to hematoxylin and eosin (HE) staining. Melanin deposition has been observed in tumors (*A*, *B*). Positive staining with HMB-45, S-100, and Melan-A of the surgical tissue (*C*, *E*, *G*) and the mouse tissue (*D*, *F*, *H*).

Subsequent to the subcutaneous implantation of OS-MM cells into the dorsal region of SCID mice, the development of tumors was observed macroscopically after a period of 6 mo (Fig. 3*C*). The histological appearance of the tumor tissue formed in nude mice showed clear features of malignant melanoma and was highly similar to the histological appearance of the patient-derived clinical specimen. In the HE-stained specimens of surgical tissue and mouse xenograft tissue, the lesions consist of fasciculated proliferation of spindle cells with marked atypia, and numerous melanin deposits are observed within the cells (Fig. 4*A*, *B*). Furthermore, in immunohistochemical staining, HMB-45, S-100, and Melan-A expressions were positive in all tissues (Fig. 4*C*–*H*). Thus, the findings derived from the analysis of tumor tissue images from patients and nude mice exhibited strong consistency.

**Figure 5.**
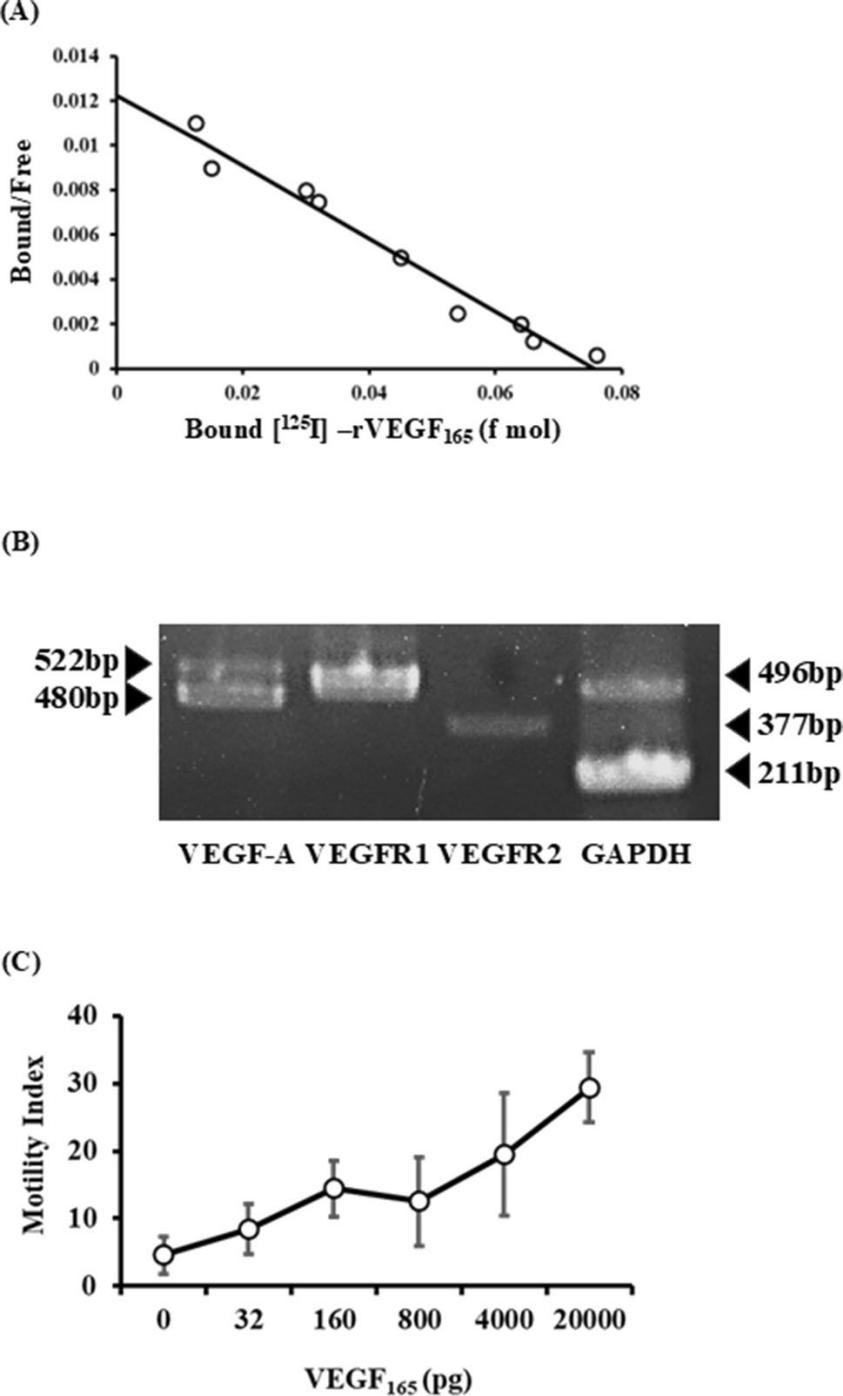
Expression of VEGF, VEGFRs and the effect of rVEGF_165_ on cell migration in OS-MM cells. Scatchard analysis of [^125^I]–rVEGF_165_ binding to OS-MM cells is shown. At 4 °C, rVEGF_165_ binding to OS-MM cells was measured under conditions where the concentration of rVEGF_165_ was incrementally increased in the presence of 100-fold excess unlabeled rVEGF_165_. The OS-MM cells express. The OS-MM cells express 1000 high-affinity receptors with a dissociation constant of 38 pM per cell (*A*). The OS-MM cells expressed the mRNAs of VEGF-A, VEGFR1/flt-1, and VEGFR2/KDR (*B*). The exogenous addition of rVEGF_165_ enhanced the motility of OS-MM cells in a concentration-dependent manner. The cell numbers in the four fields were enumerated in each of the three distinct experiments. The results were expressed as the mean number of migrating cells/mm^2^ ± standard deviation (SD), indicated as a measurement of variability (*C*).

A Scatchard plot (Scatchard 1949) of the ^125^I-VEGF binding data exhibited a straight line (Fig. 5*A*), which was consistent with a model involving one affinity class of binding sites. The high-affinity sites demonstrated a dissociation constant of 38 pM, with 1000 binding sites per cell. The mRNA expression of VEGF-A, VEGFR1/flt-1, and VEGFR2/KDR in OS-MM cells was examined using RT-PCR. The PCR products of VEGF_121_, VEGF_165_, and VEGF_189_, which are splicing variants of VEGF-A, were detected in the cells, as was the expression of VEGFR1/flt-1 and VEGFR2/KDR mRNAs (Fig. 5*B*). The motility of OS-MM cells was evaluated using the modified Boyden chamber method. The results demonstrated that a positive correlation between the concentration of VEGF_165_ and an increase in cell motility (Fig. 5*C*).

We conducted an analysis of SNVs and InDels in 88 genes, as well as fusions in 3 genes, in the OS-MM cells. The data suggest that the mutation in GNAS (NM_000516:c.602G>A; p.Arg201His) may be clinically pathogenic. The gene mutations identified in this study are shown in Table 3.

**Table 3.** Cancer panel sequencing analysis

Gene	Transcript ID	DNA change	AF (%)	Exonic effect	Clinical
	(Exon no.)	Protein change	(Alt/total)		Significance
GNAS	NM_000516	c.602G >A	31.5	Missense variant	Pathogenic
	(8/13)	p.Arg201His	(223/709)		
BRCA2	NM_000059	c.7052C > G	86.6	Missense variant	Benign/likely benign
	(14/27)	p.Ala2351Gly	(239/276)		
IDH2	NM_002168	c.939A > G	65.4	Synonymous variant	Benign/likely benign
	(7/11)	p.Gly313Gly	(530/811)		
NTRK1	NM_002529	c.1080G > A	45.5	Synonymous variant	Benign
	(8/17)	p.Thr360Thr	(121/266)		
PTEN	NM_000314	c.−366delT	100	5 prime UTR variant	Benign
	(1/9)	-	(142/142)		
PTEN	NM_000314	c.−366G > C	100	5 prime UTR variant	Benign
	(1/9)	-	(105/105)		

## Discussion

Research on permanent cell lines established from malignant tumors has played a crucial role in fostering biological understanding. Several studies have been conducted using MM cell lines derived from skin (Koizumi *et al*. 2022; Liu *et al*. 2025; Sergio *et al*. 2026). However, research utilizing MM cell lines could not be performed owing to their lack of availability. The present study reports on the successful establishment of a new MM cell line designated OS-MM. This cell line exhibits several malignant melanoma properties, including melanin production, 5-S-cyanodiphenyl-d-alanine (5-S-CD) production, tumorigenicity in SCID mice, VEGFR expression, and enhanced rVEGF_165_-mediated motility.

Mitochondrial DNA (mtDNA) typing using PCR has been widely established as a powerful tool in forensic science (Holland and Parsons 1999). This technique has been primarily applied to severely degraded specimens such as bone, teeth, and hair samples, due to its higher amplification success rate compared to nuclear DNA typing. In addition to systematic validation studies, including interlaboratory studies (Wilson *et al*. 1995; Carracedo *et al*. 1998), numerous successful identification cases have been reported (Ivanov *et al*. 1996; Lutz *et al*. 1996). The approximately 30-yr-old tissue-embedded paraffin blocks utilized in this study exhibited severe degradation, precluding the successful extraction of genomic DNA. In the mtDNA HV1 and HV2 region sequence database, genetic diversity was estimated to be 0.996% using Tajima’s method (Tajima: 1989) and 0.96% using the random match probability (RMP) approach developed by Stoneking *et al*. (1991) (Tajima 1989). In this study, the analysis of the HV1 region of mtDNA extracted from the paraffin blocks was precluded by the occurrence of mtDNA degradation.

The chromosomal composition of cancer cells is known to differ from that of normal cells and exhibits a wide range of chromosomal distributions depending on the specific type of carcinoma (Sakaki *et al*. 1971). While normal melanocytes were predominantly euploid, all melanoma cells were aneuploid, and all cell lines exhibited an average chromosome number greater than 46. The OS-MM cells exhibited a hypotriploid chromosome pattern, consistent with previous reports (Glovanella *et al*. 1976). According to Horikoshi *et al*. (1974), prolonged proliferation of cancerous cells has been shown to cause fluctuations in chromosome number, thereby resulting in transitions from diploid to tetraploid states (Horikoshi *et al*. 1974). Consequently, it was deemed imperative to periodically assess the chromosome number in the cell lines established in this study.

Melanin, a distinctive feature of malignant melanoma, is a reliable indicator of the clinical state of the disease. 5-S-CD, a metabolite of melanin, has been identified as a particularly sensitive reflection of melanoma’s progression. Consequently, it serves as a valuable indicator for the early detection of malignant melanoma and for estimating recurrence and metastasis, thereby aiding in the assessment of treatment efficacy (Yamada *et al*. 1992; Wakamatsu *et al*. 2020). A variety of immunohistochemical markers have been identified as useful tools for the diagnosis of malignant melanoma and the prediction of its prognosis. The most widely used diagnostic markers include Melan-A, HMB-45, and S-100. These models have been shown to exhibit high specificity and sensitivity (Costache *et al*. 2025). In this case as well, the expression levels of 5-S-CD, HMB-45, S-100, and Melan-A were found to be positive in all cases, consistent with previous reports (Yachida *et al*. 2011; Costache *et al*. 2025).

Advancements in the molecular analysis of melanoma have contributed to the development of novel therapeutic interventions, including BRAF inhibitors and MEK inhibitors (Davies *et al*. 2002; Chapman *et al*. 2011; Flaherty *et al*. 2012). Genetic analysis of tumor tissue is not only essential for selecting these inhibitors but also crucial for elucidating the mechanisms of melanoma carcinogenesis. The efficacy and safety of the combination therapy, dabrafenib plus trametinib, a molecular targeted drug, for unresectable advanced or recurrent melanoma with BRAF V600E mutation positivity have been demonstrated (Subbiah *et al*. 2023). Furthermore, while immune checkpoint inhibitors are the prevailing therapeutic modality for malignant melanoma that harbors NRAS gene mutations, their effectiveness in advanced malignant melanoma remains inadequate. In recent years, combination therapy involving the BRAF/CRAF inhibitor naparibinib (LXH254) and the MEK inhibitor trametinib has been reported to demonstrate promising antitumor activity in patients with NRAS mutation-positive advanced/metastatic malignant melanoma (de Braud *et al*. 2023). Consequently, we conducted NGS-based SNV/InDel (88 genes) and fusion gene (3 genes) mutation analysis in the OS-MM cells established in this study. As a result, no genetic mutations were identified in BRAF or NRAS. Preliminary data suggest that the GNAS gene mutation (NM_000516: c.602G>A; p.Arg201His) may possess clinical pathogenicity. As demonstrated in the research by Kawabata *et al*. (2022) and Mascioli *et al*. (2023), there is a correlation between mutations in the GNAS gene and diseases such as intraductal papillary mucinous neoplasms (IPMN) and McCune-Albright syndrome (Kawabata *et al*. 2022; Mascioli *et al*. 2023). The GNAS gene functions as the alpha subunit, increasing cAMP concentration by activating adenylate cyclase, thereby activating cAMP-dependent kinase (PKA) (Abbas *et al*. 2024). This pathway plays a crucial role in cell growth and differentiation. It has been reported that in metastatic lesions and in the invasive portion of melanoma patients, in addition to ERBB2 gene amplification, MET gene amplification and GNAS^R201H^ gene mutation were observed (Wang *et al*. 2024).

In our previous study, the concentration of VEGF_165_ in the culture medium of OS-MM cells was found to be 2.5 to 50 times higher than that in other skin-derived melanoma cell lines (Koizumi *et al*. 2022). Furthermore, the presence of melanoma cells expressing the VEGFR1/flt-1 and VEGFR2/KDR receptors for the angiogenic factor was observed. Gitay-Goren *et al*. (1993) also observed that melanoma cells but not melanocytes expressed receptors for VEGF (Gitay-Goren *et al*. 1993). Receptor binding assay using ^125^I-VEGF revealed that OS-MM cells express 1000 high-affinity receptors with a dissociation constant of 38 pM per cell. However, increased proliferation of OS-MM in response to rVEGF_165_ was not observed. The motility of OS-MM cells was increased by rVEGF_165_ in a concentration-dependent manner. Because OS-MM cells express VEGFRs and secrete VEGF_165_, the increase in OS-MM cell motility is likely an autocrine effect. The combination of Axitinib, a VEGFR inhibitor, and Toripalimab, an anti-PD-1 antibody, has already been reported to achieve an overall response rate (ORR) of 48.3% (95% confidence interval [CI] 29.4–67.5%) and a median progression-free survival (PFS) of 7.5 mo (95% CI 3.7–NR) (Sheng *et al*. 2019). In the future, the development of molecular targeted therapy for melanoma that targets VEGFR is anticipated.

In summary, a novel human OS-MM cell line was established and its properties were characterized. This particular cell line has been found to demonstrate melanin production capability, tumorigenicity in SCID mice, and VEGF and VEGFR1/R2 expressions. However, no BRAF or NRAS mutations were detected. The OS-MM cell line should contribute to advances in personalized cancer therapy for oral mucosal malignant melanoma.

## Supplementary Information

Below is the link to the electronic supplementary material.

MOESM1 (PDF 187 KB)

MOESM2 (JPG 532 KB)

## Data Availability

The datasets used in this study are available from the corresponding author upon request.
