# Supplementary material for: Establishment and characterization of a cell line (OS-MM) originating from a human malignant melanoma of the oral mucosa
Source: In Vitro Cell Dev Biol Anim. 2026 Feb 21;62(5):673–84. doi: 10.1007/s11626-026-01162-2 (PMC13246917; doi:10.1007/s11626-026-01162-2)
Supplement: Supplementary file 1 — (PDF 187 KB) [file 11626_2026_1162_MOESM1_ESM.pdf]

Table S1

List of analyzed genes (Axen™ Cancer Panel 1)

SNV / InDel (88genes)	Fusion (3 genes)
ABL1, AKT1, AKT3, ALK, APC, AR, ATM, AXL, BRAF, BRCA1, BRCA2, CCND1, CDH1, CDK4, CDK6, CDKN2A, CEBPA, CSF1R, CTNNB1, DDR2, EGFR, ERBB2, ERBB3, ERBB4, ERG, ESR1, ETV1, ETV4, ETV5, EZH2, FANCA, FANCC, FANCF, FANCG, FBXW7, FGFR1, FGFR2, FGFR3, FGFR4, FLT3, FOXL2, GNA11, GNAQ, GNAS, HNF1A, HRAS, IDH1, IDH2, JAK1, JAK2, JAK3, KDR, KIT, KRAS, MAP2K1, MAP2K2, MAP2K4, MET, MLH1, MPL, MTOR, MYC, MYCN, NOTCH1, NPM1, NRAS, NTRK1, NTRK2, NTRK3, PDGFRA, PIK3CA, PIK3R1, PPARG, PTEN, PTPN11, RAF1, RB1, RET, ROS1, RUNX1, SMAD4, SMARCB1, SMO, SRC, STK11, TP53, VHL, WT1	ALK, RET, ROS1
