# Supplementary figures and images for: Establishment and characterization of a cell line (OS-MM) originating from a human malignant melanoma of the oral mucosa
Source: In Vitro Cell Dev Biol Anim. 2026 Feb 21;62(5):673–84. doi: 10.1007/s11626-026-01162-2 (PMC13246917; doi:10.1007/s11626-026-01162-2)

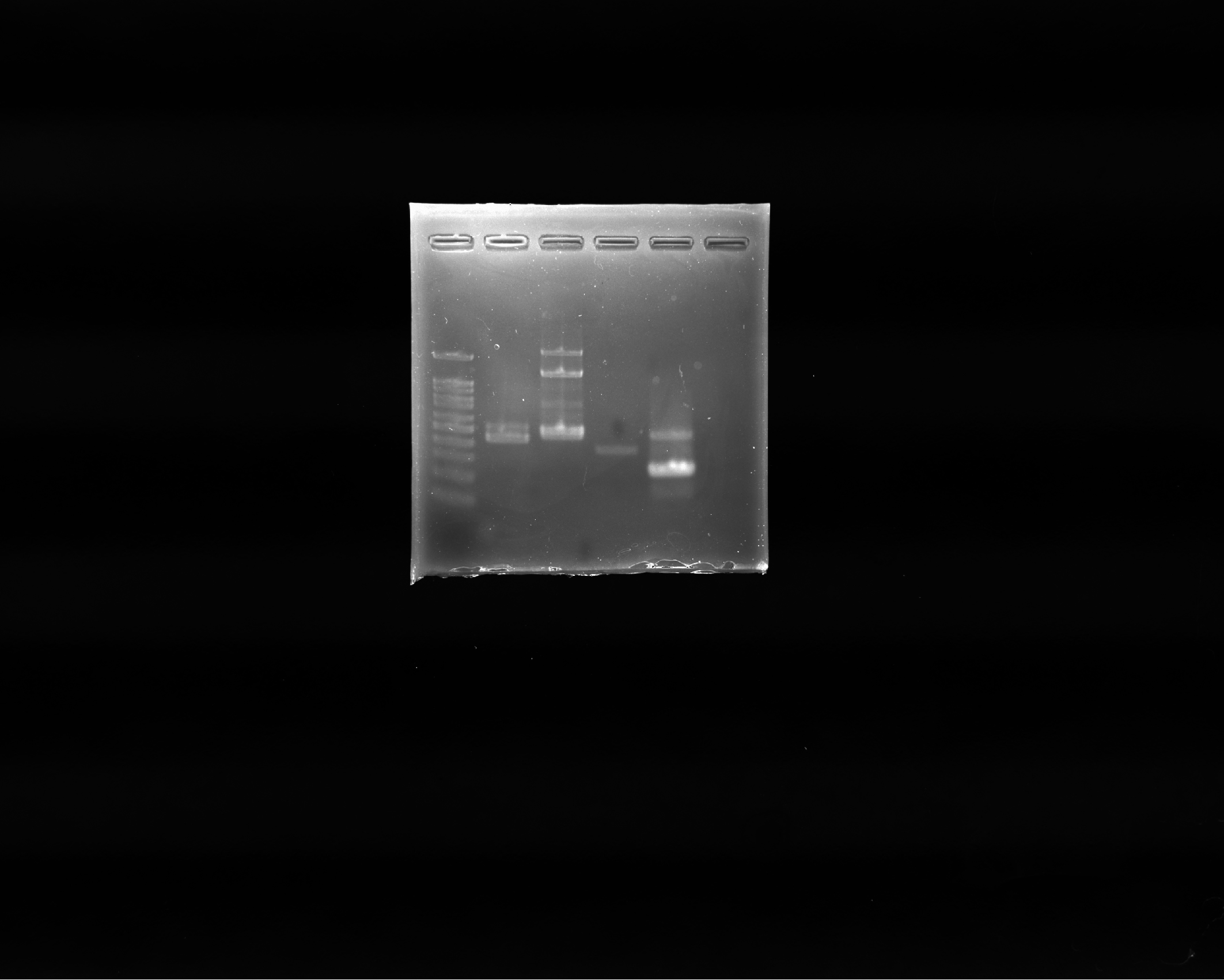

Supplement: Supplementary file 2 — (JPG 532 KB) [file 11626_2026_1162_MOESM2_ESM.jpg]
